# Natural cycle versus artificial cycle in frozen-thawed embryo transfer: A randomized prospective trial

**DOI:** 10.4274/tjod.47855

**Published:** 2018-03-29

**Authors:** Marzieh Agha-Hosseini, Leila Hashemi, Ashraf Aleyasin, Marzieh Ghasemi, Fatemeh Sarvi, Maryam Shabani Nashtaei, Mahshad Khodarahmian

**Affiliations:** 1 Tehran University of Medical Sciences Faculty of Medicine, Shariati Hospital, Department of Infertility, Tehran, Iran; 2 Zahedan University of Medical Sciences, Aliebneabitaleb Hospital, Pregnancy Health Research Center, Department of Obstetrics and Gynecology, Zahedan, Iran; 3 Tehran University of Medical Sciences Faculty of Medicine, Department of Anatomy, Tehran, Iran

**Keywords:** Frozen-thawed embryo transfer, artificial cycle, natural cycle, clinical pregnancy rate

## Abstract

**Objective:**

To investigate whether there was a difference in pregnancy outcomes between modified natural cycle frozen-thawed embryo transfer (NC-FET) cycles and artificial cycles (AC)-FET in women who all had regular menstrual cycles.

**Materials and Methods:**

One hundred seventy patients who met the inclusion criteria and had at least two cryopreserved embryos were included in a prospective randomized controlled trial. Eighty-five patients were randomized based on Bernoulli distribution into the following two groups: 1) Modified NC-FET using human chorionic gonadotropin for ovulation induction and 2) AC-FET, in which endometrial timing was programmed with estrogen and progesterone. The main studied outcome measure was the clinical pregnancy rate per cycle.

**Results:**

No significant differences were found between the two groups with regard to the chemical, clinical, and ongoing pregnancy rates (48.2% vs 45.9%, p>0.05; 38.9% vs 35.3%, p>0.05; and 37.6% vs 34.1%, p>0.05, respectively), as well as the live birth or miscarriage rates per cycle (35.3% vs 31.8%, p>0.05; and 1.2% vs 1.2%, p>0.05, respectively).

**Conclusion:**

These findings suggest that although both FET protocols are equally effective in terms of pregnancy outcomes in women with regular menstrual cycles, NC-FET is more favorable because it requires no medication, has no adverse events, and has a significant cost reduction.

**PRECIS:** Modified natural cycles are recommended in frozen–thawed embryo transfer cycles, at least in patients with regular menstrual cycles due to numerous advantages including no medication, no adverse events, and a significant cost reduction.

## Introduction

Many patients have benefited greatly from frozen–thawed embryo transfer (FET) cycles to achieve pregnancy following either successful *in vitro* fertilization (IVF) or failed fresh embryo transfer (ET) cycles^(1)^. The cost-effective FET cycles improve the cumulative pregnancy rate per oocyte retrieval^(2,3,4)^. Additionally, IVF-associated complications such as Hyperstimulation syndrome and multiple births can effectively be prevented by FET^(5)^.

An important factor in improving FET is optimal endometrial receptivity as well as synchronization between embryonic and endometrial developments^(6,7,8)^. To achieve this, several methods for endometrium preparation have been proposed. In FET cycles, the transfer of embryos may be timed either in natural cycles after spontaneous ovulation or in artificial hormonally-controlled cycles using sequentially administered exogenous estrogen (E) and progesterone^(9,10,11,12)^. Natural cycle-FET (NC-FET) may be preferable for women with regular menstrual cycles because it requires less medication and has a lower cost for patients. Nevertheless, even in these women, ovulation may not always happen or an unexpected ovulation may occur. Thus, the timing of FET can also be problematic. Furthermore, the predictability and reliability of artificial cycle-FET (AC-FET) cycles have been favored in clinics^(1,6)^. A recent systematic review and meta-analysis of the literature that compared different protocols for FET reported no differences in the clinical pregnancy rate, ongoing pregnancy rate, and live birth rate^(11)^. However, some controversy exists because some investigators reported better pregnancy outcomes with AC-FET,^(1,2,5,13)^ whereas the results of some retrospective studies demonstrated the superiority of NC-FET^(14,15)^. Because there are insufficient well-designed randomized controlled trials (RCTs) to determine which type of cycle regimen is superior in FET cycles^(6,12)^ and considering the existence of conflicting reports in this regard, this study focused on comparing two different protocols for endometrial preparation: modified NC-FET versus AC-FET in women who all had regular menstrual cycles. The chemical, clinical, and ongoing pregnancy rates, miscarriage and live birth rates were compared in these two distinct FET cycles to identify predictive factors and to influence the reproductive outcome.

## Materials and Methods

**Figure 1:**
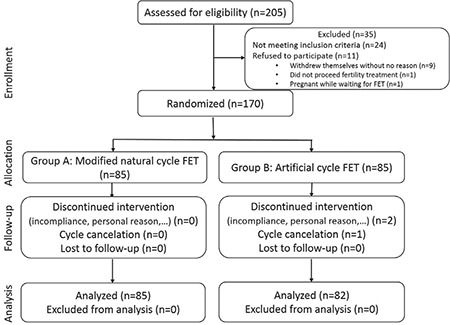
Participant consolitated standards of reparting trials flow diagram *FET: Frozen-thawed embryo transfer*

After obtaining institutional review board approval, this prospective randomized clinical trial was approved by the Ethics Committee of Tehran University of Medical Sciences (approval number: IR.TUMS.REC.1394.2051) and written informed consent was obtained from all participants. All women who were aged between 18 and 40 years and had regular menses (25-34 days) and who had at least two cryopreserved embryos derived from intracytoplasmic sperm injection (ICSI) treatment cycles from January 2012 to December 2014 were referred to the infertility clinic of Shariati Hospital (a university teaching hospital) and were enrolled in the study. The period of study was from January 2015 to July 2016. Women with endometriosis, immune diseases, recurrent abortion, donated sperm or oocyte, uterine abnormality, ovarian cyst or previous ovarian surgery, history of previous IVF failure, and any known contraindications or allergy for oral estradiol or progesterone therapy were excluded from participating in the study. In addition, patients were excluded if their clinical history included percutaneous epididymal sperm aspiration or testicular sperm extraction. The enrolled women were divided randomly into two groups to undergo either a modified natural cycle FET (group A) or artificial cycle FET (group B) using computerized software in a 1:1 fashion (Figure 1). The sequence of allocation to the two groups was generated by the aforementioned software and then the treating physicians (n=2) gave treatment based on the allocated chart. A baseline transvaginal ultrasound (Siemens, Sonoline G20) using a 7.5 MH transvaginal probe was performed in all patients by the same attending physician on the 2^nd^ or 3^rd^ days of the menstrual cycle to assess the endometrium and rule out the presence of an ovarian cyst.

### The endometrial preparation protocols

Based on the study protocol and inclusion criteria, 85 patients were considered as group A (modified NC-FET) and 85 were classified as group B (AC-FET) and were assigned to receive the related protocol. The demographic characteristics and clinical data of the two groups are given in Table 1. 

### Modified natural cycle frozen–thawed embryo transfer

An ultrasound examination was performed on days 10 to 12 of the cycle after a spontaneous menses to detect the leading follicle. When at least one dominant follicle reached ≥18 mm in diameter and the thickness of the endometrium was at least 8 mm, a bolus of 10.000 IU of human chorionic gonadotropin (hCG) (Pregnyl; N.V. Organon, Oss, The Netherlands) was injected intramuscularly for the induction of ovulation and the embryos were thawed and transferred 4 days later.

### Artificial cycle frozen–thawed embryo transfer

From the 21^st^ day of the previous cycle, 500 µg/day of buserelin acetate (Suprecur; Hoechst UK Ltd, Hounslow, UK) was subcutaneously injected. Oral estradiol valerate (Progynova, Bayer, Germany) was then administered from day 2 of the next cycle from 2 mg/d to 2 mg/d Ч4. The E dosage was adjusted based on the endometrial thickness as assessed using transvaginal ultrasound. After a baseline transvaginal ultrasound, a second ultrasound examination was performed on days 10 to 12 for the endometrial thickness assessment. Four hundred milligrams Ч2 daily progesterone vaginal suppositories (Cyclogest, Actavis, Devon, UK) were administered in the following 3 days when the endometrium reached a thickness of 8 mm or maximum. ET was performed after 3 days of progesterone administration. Luteal phase support commenced on the day of ET in all the participants, using 400 mg Ч2 daily progesterone vaginal suppositories (Cyclogest, Actavis, Devon, UK). Serum beta-hCG levels were evaluated for all patients 14 to 16 days after ET to confirm biochemical pregnancy. Progestin support continued up to the end of the 12^th^ weeks’ gestation if pregnancy was achieved. Vitrification and thawing of the cleavage-stage embryo were implemented using the same method reported in a previous publication^(16)^. The embryos were thawed on the day of the ET and those that were classified as grade I or grade II (according to cleavage stage, blastomere size and shape, and fragmentation) and had at least 50% intact blastomeres were transferred. The number of transferred embryos per cycle was limited to a maximum of three and was dependent upon the number of previous treatments, the number of embryos frozen in the same straw, and the quality of available embryos. Moreover, similar techniques were used by the two expert physicians who performed ET. 

### Outcome measures

The primary outcome measure was clinical pregnancy. The secondary outcomes were biochemical pregnancy, ongoing pregnancy, live birth rate, and miscarriage rate. We applied the term “biochemical pregnancy” to an elevated serum beta-hCG level two weeks after hCG administration. Clinical pregnancy was established by the detection of a fetal heartbeat through transvaginal ultrasound in the 6^th^ week. “ongoing pregnancy” referred to any pregnancy beyond 20 weeks of gestation. The miscarriage rate was measured using transvaginal ultrasonography and a decrease in serum beta-hCG level. All pregnant women were followed up to obtain delivery data. A live birth was defined as the completion of expulsion or extraction of a live baby from its mother. 

### Statistical Analysis

To have power of 0.8 to detect a 10% difference in clinical pregnancy rates between the study groups with a significance level of 0.05, we required 80 patients in each study group. Data are expressed as mean ± standard deviation for descriptive statistics when normally distributed, otherwise as median and range. All the analyses were performed using the SPSS software (version 17.0 for Windows; SPSS Inc., Chicago, IL, USA). The normality of distribution was checked using the Kolmogorov-Smirnov test. The comparison of the treatment outcomes between the two protocols was performed using the independent Sample t-test and/or chi-square test (or Fisher’s exact test if required). The level of significance was p<0.05. 

## Results

A total of 205 women undergoing ICSI who had cryopreserved embryos were evaluated, 170 of whom were randomized to receive either modified NC-FET cycles or AC-FET (Figure 1). There were no statistically significant differences between the two groups terms of age, duration, type, and causes of infertility, day 3 follicle stimulating hormone or body mass index (Table 1). The characteristics and pregnancy outcomes for both cycle types are shown in Table 2. No significant difference was found between the NC-FET and the AC-FET groups regarding the average number of dominant follicles, endometrial thickness, the average number of transferred embryos, and embryo grade. Of the 170 cycles, a total of 63 clinical pregnancies occurred in the NC-FET and the AC-FET groups [33 (38.9%) versus 30 (35.3%) clinical pregnancies; p=0.4, respectively]. As demonstrated in Table 2, there were no significant differences between the two cycle types in terms of chemical, clinical, and ongoing pregnancy rates, miscarriage and live birth rates.

## Discussion

This prospective RCT demonstrated that there were no differences in FET outcomes between modified natural and artificial cycles in patients with regular menstrual cycles. In the present study, patients in the modified natural cycle group received supplemental hCG (as a trigger to ovulation) and transvaginal progesterone to offset any probability of poor endogenous luteal phase. Patients with artificial cycles depended entirely on exogenous estradiol and progesterone, with prior gonadotrophin-releasing hormone agonist (GnRHa) down-regulation. The investigation of factors that affect the success of FET has steadily intensified during the past few years in order to transfer fewer embryos and to improve laboratory techniques^(12,17,18,19)^. The success of FET is dependent upon the reciprocal interaction between embryo development and the receptive uterus,^(5,7)^ which can be evaluated through endometrial volume, endometrial thickness, and artery blood flow^(8)^. Numerous biomarkers, including leukemia inhibitory factor, integrin, and homebox A10^(20,21,22)^ have been proposed as reliable markers of a receptive endometrium. Moreover, in older women, endometrial development in the follicular phase can be negatively affected by age, resulting in a lower pregnancy rate^(12)^. Adequate endometrial development in FET cycles can be achieved through three frequently used cycle regimens: natural cycles with or without ovulation induction using hCG; hormonally-manipulated artificial cycles using E followed by progesterone to prime the endometrium with/without a GnRHa; and stimulated cycles in which follicular development is supported by follicle-stimulating hormones^(15)^. However, no consensus has yet been reached regarding the best FET protocol for endometrial preparation^(2,15)^. Zheng et al.^(5) ^reported that ovulation in hormone replacement treatment (HRT) cycles had a detrimental effect on pregnancy, although HRT increased the possibility of pregnancy. This finding is in line with the results of other large retrospective studies^(1,13)^ that also reported a higher positive pregnancy test rate in the substituted cycle with E and progesterone than in natural cycles with hCG or progesterone. In contrast, some studies reported superior pregnancy outcomes in natural cycles^(14,15)^. Higher estradiol (E_2_) levels may interfere with the window of implantation and cause endometrial receptivity and implantation windows to close earlier^(23)^. Fritz et al.^(24)^ also suggested that elevated E_2_ levels were associated with lower ongoing pregnancy/live birth rates, possibly due to the opposing effect on the endometrium from excess unopposed E_2_ exposure. Furthermore, it has recently been reported that natural cycles have a better effect on endometrial transcriptome than artificial cycles in which E has a stronger negative effect than progesterone on the endometrial transcriptome^(25)^. On the other hand, consistent with our findings, comparable FET outcomes have been suggested by several studies in natural and artificial FET cycles^(12,19,26,27,28,29)^. In addition, a 2017 update of the 2008 Cochrane review also showed no evidence of a difference between the two cycles in rates of live birth or miscarriage rates^(12,30)^. AC-FET can be more easily scheduled, which leads to a better control of embryo thawing and transfer timing and also decreases cancellation rates compared with NC-FET. This is the result of ovulation suppression and the programmed replacement of exogenous hormones^(5)^. However, these advantages are somewhat counterbalanced by its possible adverse effects through exposure to exogenous hormones, higher risk of thrombo-embolic events, and providing a higher financial burden on patients, a burden that many are incapable of overcoming^(12,31,32)^. Although NC-FET is complicated to plan due to its requirement for more frequent ultrasonographic evaluations of the dominant follicle, the risk of unexpected ovulation and insufficient development of the endometrium, its advantages such as being more patient friendly, convenience, less medication, and lower price cannot be denied^(6,28)^. Consequently, patients should be given the option of NC-FET in order to maintain autonomy in choosing the cycle protocol.

### Study Limitations

Further clinical trials with larger sample sizes are required to illuminate the clinical and biochemical benefits of NC-FET. 

## Conclusion

In conclusion, based on the results of our study, modified natural cycles should be recommended in FET because they carry numerous advantages and have comparable FET outcomes, it at least in patients with regular menstrual cycles. 

## Figures and Tables

**Table 1:**
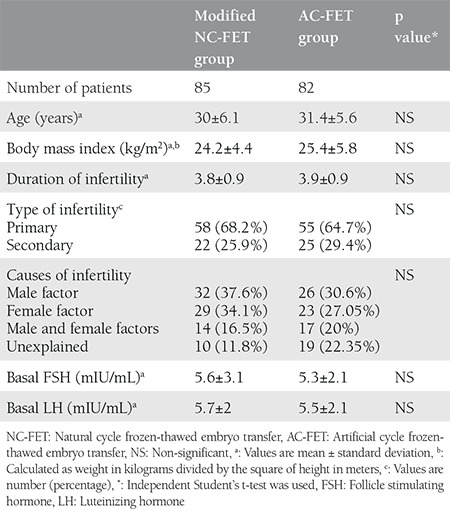
Demographic characteristics and clinical data of modified natural cycle frozen-thawed embryo transfer and artificial cycle-frozen-thawed embryo transfer groups

**Table 2:**
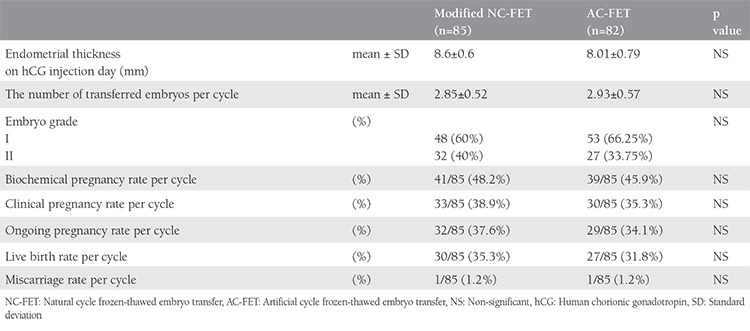
Characteristics and pregnancy outcomes of frozen-thawed embryo transfer cycles
